# Supplementary material for: High-resolution ex vivo nanoCT reveals 3D architecture of the adult male mouse lower urogenital tract
Source: PLoS One. 2025 Sep 18;20(9):e0326004. doi: 10.1371/journal.pone.0326004 (PMC12445489; doi:10.1371/journal.pone.0326004)
Supplement: S1 Table — NanoCT acquisition parameters used for imaging whole mouse urogenital tracts and microdissected urethra specimens. Scans were performed using either the v|tome|x M or nanotom M systems. Key parameters include voltage, current, filter material, voxel size, exposure settings, and total number of projection images collected per 360° rotation. (DOCX) [file pone.0326004.s001.docx]

| **S1 Table: NanoCT scanning parameters** | | |
| --- | --- | --- |
| **Parameter** | **Whole Urogenital Tract Specimen** | **Microdissected Urethra Specimen** |
| System | v\|tome\|x M | nanotom M |
| Voltage (kV) | 100 | 65 |
| Current (μA) | 100 | 250 |
| Filter | 1.0 mm copper | 0.254 mm aluminum |
| Spot Size | 0 | 0 |
| Voxel size (μm) | 11.39 | 3.5 |
| Exposure time (ms) | 500.1 | 500 |
| Frames averaged | 3 | 3 |
| Frames skipped | 1 | 1 |
| Rotation | 360° | 360° |
| Number of images collected | 2200 | 2000 |
