# Supplementary material for: High-resolution ex vivo nanoCT reveals 3D architecture of the adult male mouse lower urogenital tract
Source: PLoS One. 2025 Sep 18;20(9):e0326004. doi: 10.1371/journal.pone.0326004 (PMC12445489; doi:10.1371/journal.pone.0326004)
Supplement: S2 Table — Interclass Correlation Coefficients (ICC) with corresponding p-values assessing inter-rater reliability for quantitative measurements of the mouse prostatic urethra. ICC values were calculated from three independent raters. Interpretation thresholds are as follows: < 0.5 = poor, 0.5–0.75 = moderate, 0.75–0.9 = good, and >0.9 = excellent reliability. (DOCX) [file pone.0326004.s002.docx]

| **S2 Table. Interclass Correlation Coefficient (ICC) to assess inter-rater reliability** | | |
| --- | --- | --- |
|  | **ICC** | **p value** |
| Urethral lumen area | 0.990 | 1.39e-06 |
| Urethral lumen diameter | 0.786 | 9.77e-03 |
| Urethral lumen perimeter | 0.989 | 1.38e-06 |
| Rhabdosphincter thickness | 0.610 | 1.22e-02 |
| Prostatic duct count | 0.676 | 1.27e-02 |
| Prostatic urethra length | 0.942 | 1.34e-05 |
