# Supplementary material for: High-resolution ex vivo nanoCT reveals 3D architecture of the adult male mouse lower urogenital tract
Source: PLoS One. 2025 Sep 18;20(9):e0326004. doi: 10.1371/journal.pone.0326004 (PMC12445489; doi:10.1371/journal.pone.0326004)
Supplement: S3 Table — Dice Coefficients (DC) quantifying inter-rater segmentation variability for selected anatomical structures within the mouse lower urogenital tract. The DC measures spatial overlap between segmentations performed by independent raters, where 0 indicates no overlap and 1 indicates perfect overlap. (DOCX) [file pone.0326004.s003.docx]

| **S3 Table: Dice Coefficients (DC) to assess segmentation inter-rater variability** | |
| --- | --- |
|  | DC |
| Urethral lumen | 0.9523 |
| Seminal vesicles | 0.9203 |
| Ductus deferens | 0.9806 |
| Anterior prostate ducts | 0.9558 |
| Ventral prostate ducts | 0.8820 |
| Dorsolateral prostate ducts | 0.7945 |
| Rhabdosphincter | 0.9626 |
